# Associations between cortisol awakening response and resting electroencephalograph asymmetry

**DOI:** 10.7717/peerj.7059

**Published:** 2019-06-03

**Authors:** Hongxia Duan, Huihua Fang, Yuling Zhang, Xia Shi, Liang Zhang

**Affiliations:** 1 Center for Brain Disorder and Cognitive Science, Shenzhen Key Laboratory of Affective and Social Cognitive Science, Shenzhen University, Shenzhen, Guangdong, China; 2 Zaoyang First People’s Hospital, Zaoyang, Hubei, China; 3 Department of Psychology, Tianjin University of Technology and Education, Tianjin, China; 4 Key Laboratory of Behavioral Science, Institute of Psychology, Chinese Academy of Sciences, Beijing, China

**Keywords:** Cortisol awakening response, EEG, Alpha, Asymmetry

## Abstract

The cortisol awakening response (CAR), a rapid cortisol rise in the morning after awakening, has been proposed to provide energy to cope with daily demands and suggested to be associated with brain functions. Electroencephalogram (EEG) asymmetry studies have implicated asymmetric cortical activation, especially in frontal cortex, in approach-withdrawal motivation. In this study, we examined the relationship between the CAR and lateralized cortical activity under rest in 55 university male students. Saliva samples were collected at 0, 15, 30 and 60 min after awakening on the two consecutive workdays. The lateralized cortical activity at frontocentral sites was examined by alpha asymmetry score. The results showed that a higher CAR was positively associated with alpha asymmetry score, which indicated that the higher CAR is linked with more left-sided cortical activity at frontocentral sites under resting state. This association still existed even after controlling psychological and sleep quality variables. These results suggested that appropriately mobilizing energy resource storage after awakening revealed as CAR might be associated with goal-directed approach tendencies before any eventual stressful situation, characteristic of more left than right resting-state frontocentral cortical activity.

## Introduction

The activity of the hypothalamus-pituitary-adrenal (HPA) axis has a marked diurnal circadian rhythm, which is characterized by the levels of its end-product cortisol in human. Cortisol levels increase rapidly within the first 20–30 min after awakening, then return to baseline levels about 1 h later. This phenomenon, known as cortisol awakening response (CAR), is consistent across consecutive days (Pruessner et al., 1997) and has good intra-individual stability by genetic influences (Wust et al., 2000).

The functional role of the CAR has been proposed to provide energy resources to the transition from sleep to wakefulness and to deal with anticipatory demands of the upcoming day (Clow et al., 2004; Fries, Dettenborn & Kirschbaum, 2009). This interpretation is consistent with several lines of evidence which on one hand showed that the higher CAR was associated with perceived workload (Steptoe et al., 2000), and the higher CAR in weekday compared to weekend (Thorn et al., 2006) and on competition day compared to normal control day (Rohleder et al., 2007). On the other hand, the decreased CAR has been observed in individuals with fatigue, exhaustion and burnout (Nater et al., 2008; Pruessner, Hellhammer & Kirschbaum, 1999; Sonnenschein et al., 2007). A recent study showed that children from low socioeconomic status also had decreased CAR, which was mediated by parental and child anxiety (Zhu et al., 2019). This bidirectional association might suggest the role of coping style in the CAR, that is, the experience of general and work stress elicits an active engagement with the environment and the higher CAR, while prolonged stress and burnout-related factors elicit conservation/withdrawal response with the environment and the lower CAR (Chida & Steptoe, 2009).

Brain regions including hippocampus, amygdala and the prefrontal cortex (PFC) are implicated in the regulation of the HPA axis including CAR (Buchanan et al., 2004; Clow et al., 2010; Fries, Dettenborn & Kirschbaum, 2009). Alterations in the CAR were also found to be associated with these regions-supported cognition and emotion. For example, an elevated CAR linked with better frontal cortex-dependent working memory (Almela et al., 2012) and executive function (Evans et al., 2012). The higher CAR was also associated with less aggressive behavior (Bohnke et al., 2010), more problem-focused coping (Gilbert et al., 2017) and better regulation of self-related negative information implemented by dorsal ACC (Kuehner, Holzhauer & Huffziger, 2007; Quevedo et al., 2017). Furthermore, high CAR was a moderator of the relationship between acute stress disorder symptoms after workplace violence exposure and subsequent posttraumatic stress symptoms, which suggests that higher CAR is a protective factor in that it abolishes the link between acute trauma and subsequent posttraumatic stress symptoms (Marin et al., 2019).

The neural mechanism underlying the relationship between CAR and cognition/emotion has not been fully elucidated. Evidence from stress- or drug-induced cortisol showed that several hours after cortisol peak, slow corticosteroid effects develop to restore homeostasis and bring back PFC functions to enhance working memory, promote sustained attentional processing and reduce amygdala activity by long-lasting genomic corticosteroid actions (Henckens et al., 2010, 2011, 2012; Joels et al., 2008). Human rapid transcranial magnetic stimulation (rTMS) study found that days with larger than individual average CARs are associated with greater rTMS-induced synaptic plasticity in motor cortex (Clow et al., 2014). With the rodent model, Liston et al. (2013) further showed that circadian glucocorticoid peaks promote synapse formation in the mouse cortex after motor skill learning through both non-transcriptional and transcriptional mechanisms.

The characteristic of emotion processing has also been extensively investigated by lateralized cortical activity, that is the left hemisphere, especially the frontal region that is involved in positive emotion processing, active coping and approach-related motivation, whereas the right hemisphere is more involved in negative emotion processing, punishment and withdrawal-related motivation (Davidson, 1992, 1995; Coan & Allen, 2004). Electroencephalogram (EEG) alpha asymmetry in resting state has long been used to measure this lateralized cortical activation (Coan & Allen, 2004; Reznik & Allen, 2018). Under resting state, the alpha asymmetry in frontal regions reflects endogenous cortical activity which is relatively stable over measurements, therefore, it may serve as an endophenotype for normal as well as abnormal emotion processing (Allen & Cohen, 2010). Multiple lines of evidence have shown that individuals with greater right than left frontal activity in baseline report higher level of negative affect and greater negative response to negative emotion-inducing stimuli and smaller positive response to positive emotion-inducing stimuli (Tomarken, Davidson & Henriques, 1990; Tomarken et al., 1992; Wheeler, Davidson & Tomarken, 1993). In the same vein, greater left than right frontal activity in the baseline is associated with relatively faster recovery following an aversive event (Wheeler, Davidson & Tomarken, 1993), better cognitive emotion regulation ability (Jackson et al., 2000) and higher levels of psychological well-being (Urry et al., 2004).

According to our knowledge, there was only one study exploring the relationship between the CAR and frontal asymmetry activity, which showed that a higher CAR was related to a greater right than left frontal cortical activity (Hewig et al., 2008). However, they collected only 1-day CAR. The use of CAR from a single day may produce inconsistent results because of unstable confounding factors which might have a large influence on cortisol outcomes, while the CAR of at least 2 days can achieve reliable trait measures (Hellhammer et al., 2007). Furthermore, they didn’t control any sleep and stress variables, although their participants were under examination stress. It has been demonstrated that sleep-related and psychosocial variables such as stress have a very important influence on the CAR (Fries, Dettenborn & Kirschbaum, 2009; Vargas & Lopez-Duran, 2014).

The aim of the present study was to further investigate the relationship between the CAR as a trait and alpha asymmetry score at the frontal region in a resting state. The high cortisol in the morning was proposed to lead to exploratory behavior and the facilitation of typical behavioral patterns in order to survive (Korte et al., 2005). Previous results have suggested that the higher CAR might be associated with more adaptive coping strategies to negative events or challenge (Bohnke et al., 2010; Chida & Steptoe, 2009), and relatively left frontal cortical activity underlies more active engagement coping style (Davidson, 1992, 1995). Therefore, we hypothesized in the present study that a higher CAR might be associated with a relatively left frontal activity.

## Materials and Methods

### Methods

#### Participants

This study was from a larger project which addressed the relationship between cortisol and cognition/behavior (Duan et al., 2013; Zhang et al., 2015). Considering the sex difference on the diurnal cortisol level (Miller et al., 2016), only males were recruited for this study through an advertisement in a Chinese medical college. Due to the potential impact on the HPA axis and brain function, the following exclusion criteria were included: any history of physiological, neurological or psychiatric disorders; chronic use of any psychiatric, neurological or endocrine medicine; current acute inflammation or allergy or episodes of chronic disease; irregular circadian rhythm; excessive alcohol consumption (more than two alcoholic drinks daily) and nicotine consumption (more than five cigarettes daily); any history of serious head trauma; any medication use within 2 days of participation in the study. All participants were further screened by using the life events scale (Tennant & Andrews, 1976; Zhang et al., 1987) in order to exclude participants who had experienced any major stressors during the past month. As a result, a total of 63 young healthy male college students were recruited. All these participants were in school at the time of participating in our experiment and were experiencing their regular course study. The data from eight participants were discarded because of too few available trials from artifacts (see *EEG recording and preprocessing* part for detail). Thus, the data from 55 participants (mean age 22.46 ± 0.93 years) were reported in the final analysis. Among these 55 participants, 34 participants were preparing for an examination which began 11–25 days later, and 21 participants were not. The variables of exam status, subjective stress and diurnal activity change (sleep) were taken into account in the analyses of the current study (see details below).

#### Procedures

Upon arrival in the lab, the participants first completed questionnaires including Spielberger State-Trait Anxiety Inventory (STAI) (Spielberger et al., 1983) and Perceived Stress Scale (10-item version, PSS10) (Cohen & Williamson, 1988) (see details below). Then they were seated in a sound-attenuated room in a relaxed position to be prepared for the EEG recording. After the application of the EEG map, the resting state EEG was recorded during four 1-min periods, which was the minimum to record the resting state alpha wave as the previous studies suggested (Doktor & Bloom, 1977; Goodman et al., 2013), and two were recorded with eyes open (O) and two with eyes closed (C). The orders of eyes-open and eyes-closed conditions were counterbalanced (OCCO or COOC) and were randomly assigned to each participant. The participants were instructed through a microphone when to open and close their eyes. They were told to try to relax as much as possible without falling asleep. EEG recording of this resting state was initiated by the experimenter. After the resting EEG, the participants completed other cognitive tasks which were reported elsewhere (Duan et al., 2015; Wu et al., 2014). After the experiment, participants were instructed in person about how to collect the saliva over the next two consecutive days and they also received a detailed instruction packet to remind them correctly and precisely collecting their morning saliva. During the 1 h of saliva sampling, the sleeping status questionnaire including the awakening time, sleep duration and sleep quality were completed.

#### Questionnaires

PSS10 was used to assess the long-term psychological stress (Cohen & Williamson, 1988). The state inventory from STAI assesses anxiety at the time of testing, and the trait inventory from STAI assesses trait anxiety (Spielberger et al., 1983). Sleeping status, including two parts, was administrated by a sleeping questionnaire during the saliva collection period. The first part was about awakening time and sleep duration, which was averaged between the two saliva sampling days. The second part rated the sleep quality by asking seven items: (1) how did you sleep last night (from very poorly 1 to 5, very well); (2) did you feel refreshed upon waking (from not at all 1 to 5 completely); (3) how deeply did you sleep last night (from very lightly 1 to 5, very deeply); (4) did you sleep for the entire time allocated for sleep (from woke up much earlier 1 to 5, slept for the whole night); (5) how easy was it for you to wake up (from very easy 1 to 5, very difficult); (6) how easily did you fall asleep last night (from very easily 1 to 5, very difficult); and (7) how many dreams did you have last night (from no dreams 1 to 5, many dreams). The scores of item 6 and 7 were inverted, and sleep quality was estimated by adding scores from these seven items on each night and then averaging the total scores of two nights. Thus, the higher average total score indicates better sleep quality.

### EEG recording and preprocessing

A total of 64 cap-mounted Ag/AgCl electrodes arranged according to the International 10–20 system (NeuroScan Inc., El Paso, TX, USA) were applied and EEG data were continuously recorded. On-line reference to the left mastoid and offline algebraic re-reference to the linked mastoid was used. Electrode impedance was below five KΩ, and the EEG was amplified with a bandpass filter of 0.05–100 Hz with a sampling rate of 500 Hz. Bipolar horizontal electrooculogram (EOG) was recorded from electrodes placed at the outer canthus of each eye, and bipolar vertical EOG was recorded from electrodes located above and below the left eye.

The EEG data were processed offline by Neuroscan 4.3 software. The data were low-pass filtered with a cutoff frequency of 30 Hz. Ocular artifacts were removed by a regression procedure built into the Neuroscan software combined with visual inspection and manual operation. Artifacts were rejected automatically if the signal amplitude exceeded ±100 μV. Before the spectral analysis, EEG signals were segmented into 2 s epochs. The artifact-free epochs were extracted through a Hanning window. Spectral power (μV^2^) was estimated by a fast Fourier transform in every 2 s-bin epoch and then converted to power density values (μV^2^/Hz) in the range of alpha band (8–13 Hz). For every 1-min resting EEG, only data more than 20 epochs of 2 s-bin were included in the final analysis (Lewis, Weekes & Wang, 2007). Eight participants were excluded because of less than 20 epochs. Power density values were averaged across all epochs of each 1-min recording. Then, the averaged power density values were log-transformed to normalize the distribution. In the end, power density from the four 1-min recording was averaged to get a trait-like measure of alpha asymmetry.

According to a previous study, asymmetry scores at pairs of channels around fronto-central sites showed good internal consistency and test-retest stability (Allen et al., 2004). Therefore, analyses for the present study focused on a specific subset of the frontocentral sites (FC1 & FC2, FC3 and FC4) which also corresponded to previous studies (Mennella et al., 2015; Urry et al., 2004). Power in left and right-sided electrodes was averaged first, then the alpha asymmetry scores were computed by the mean in power density of electrodes in the right hemisphere minus the mean in the left hemisphere (log-right minus log-left alpha power density). Since alpha power is inversely related to cortical activity (Pfurtscheller, Stancak & Neuper, 1996), positive difference scores indicate relative left-hemisphere activity, and negative difference scores indicate relative right hemisphere activity.

To test if the relationship between the CAR and brain activity is specific to the alpha band, we also explored the δ wave (1–3.5 Hz). The δ activity is the main characteristic of slow wave sleep or resting state, while highest δ activity is mainly observed during mental tasks in central and frontal regions. Power increases of δ frequencies is suggested to be associated with a wide range of cognitive function including attention, salience detection and functional cortical inhibition of sensory input to maintain internal concentration (for reviews, see Harmony, 2013; Knyazev, 2012). According to previous researches, δ power from Fz, FCz and Cz electrodes were extracted (Stefanics et al., 2010).

### Salivary cortisol sampling and analysis

Saliva was collected using the Salivette sampling device (Sarstedt, Germany). On two consecutive workdays, participants were instructed to collect their saliva samples immediately after awakening (S1) and 15 min (S2), 30 min (S3) and 60 min (S4) after awakening. A detailed description of the cortisol sampling can be found in other publication on this sample (Duan et al., 2013). In short, participants were asked to wake up between 06:00 and 08:00 on both days and to refrain from eating and drinking, and not to brush teeth before completing all the saliva sampling. The participants were asked to record the sampling time of each saliva sample to make sure that they adhere to the timing protocol. The experimenter explained to each participant the importance of adhering to the sampling timeline. Furthermore, each participant took a copy of the sampling instruction to increase compliance. After sampling, participants dropped off the saliva tubes to the laboratory, where the salivettes were immediately kept frozen at −20 °C until assay.

Saliva samples were thawed and centrifuged at 3,200 rpm for 10 min. Cortisol concentration was analyzed by electrochemiluminescence immunoassay (Cobas e 601; Roche Diagnostics, Numbrecht, Germany).

Since the cortisol levels at each sampling point between the 2 days were no different from paired *t*-tests (*p_s_* > 0.1) and significantly correlated (*r_s_* = 0.32–0.54, *p_s_* = 0.00–0.02), thus cortisol levels were averaged across the 2 days to increase the reliability. Two scoring methods were used to calculate CAR: (1) AUCg = (S (sample) 1 + S2) × 0.25/2 + (S2 + S3) × 0.25/2 + (S3 + S4) × 0.5/2), which represents the total cortisol secretion in 1 h after awakening; (2) AUCi = AUCg—S1 × (0.25 + 0.25 + 0.5), which represents the dynamic change in cortisol in 1 h after awakening (Hellhammer et al., 2007; Pruessner et al., 2003). Since the cortisol data is not normally distributed, a natural log transformation was applied to these cortisol data.

### Correlation analysis between CAR and EEG activity

Statistical analysis was performed by SPSS version 23 (IBM Inc., Armonk, NY, USA). Pearson correlation was conducted first to explore the possible relationship between cortisol (AUCg or AUCi) and EEG activity (alpha asymmetry scores (log-right minus log-left) and δ power). Furthermore, partial correlations were conducted to examine the relationship between the cortisol (AUCg or AUCi) and EEG activity with exam status and psychological (PSS10, state anxiety, and trait anxiety) as well as sleep-related variables (awakening time, sleep duration and sleep quality) controlled to exclude their confounding influences. Moreover, considering that Bootstrap method provides better control over type I error as well as better representation of the probability distribution which makes it a robust statistical test (Wilcox, 2011), therefore, we performed partial correlation with bias-corrected bootstrap (1,000 samples) to improve the robustness of our inference. Results are described as partial correlation with 95% confidence interval (CI) and standard errors (SE) from the bootstrap analysis. All hypotheses were examined using a two-tailed test (*p* < 0.05).

### Ethics

All the participants gave written informed consent and were paid for their participation. This experiment was approved by the Ethics Committee of Human experimentation at the Medical Department of Shenzhen University. The experiment was conducted in accordance to relevant guidelines and regulations.

## Results

### Descriptive data

The mean PSS10 score was 16.29 with standard deviation (SD) of 3.71. The mean state anxiety score before the experiment was 28.38 (SD: 7.02), and mean trait anxiety score was 32.38 (SD: 6.53). On average, participants slept 7.23 h (SD: 0.87 h) and woke up at 7:12 (SD: 34.51 min), and their sleep quality score was 24.73 (SD: 3.31).

### Cortisol level

There was a clear pattern of dynamic secretion of morning cortisol: the cortisol levels increased from awakening point (14.315, SE: 0.968 nmol/L) to peak at 15–30 min after awakening (16.706 ± 1.016 nmol/L and 16.915 ± 0.876 nmol/L, respectively), and then declined at 60 min after awakening (12.889 ± 0.583 nmol/L). The mean AUCg was 15.5312 ± 0.734 nmol/L, and the mean AUCi was 1.216 ± 0.61 nmol/L.

### Association between the cortisol level and EEG activity

The Pearson correlation analysis revealed that AUCg was marginally associated with alpha asymmetry score at frontocentral sites (*r* = 0.250, *p* = 0.065). No significant correlation was found between AUCi and alpha asymmetry score at frontocentral sites (*r* = −0.103, *p* > 0.05).

**Figure 1:**
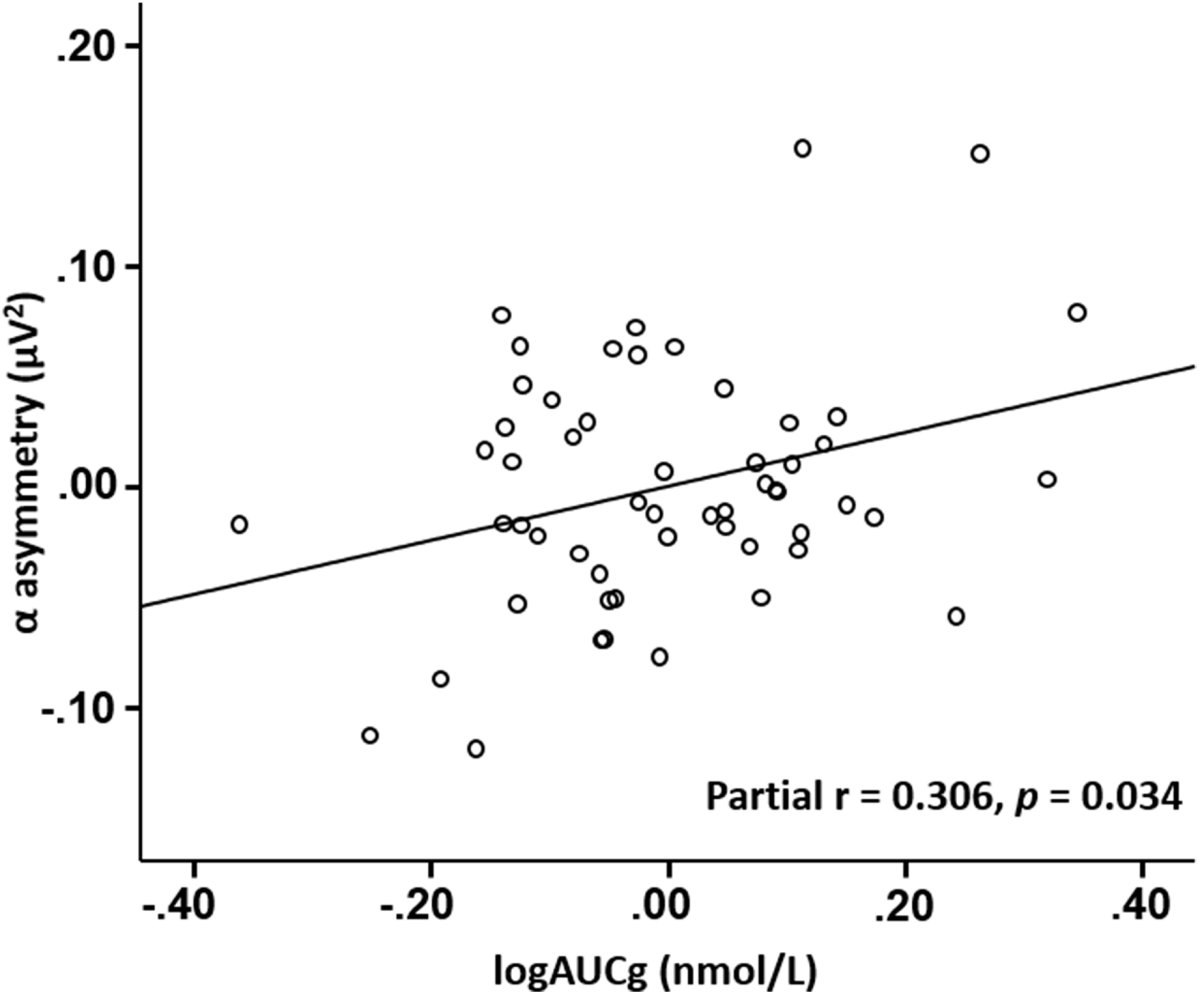
Scatter plot of partial correlation between CAR (AUCg) and alpha asymmetry score at frontocentral sites.

Bias-corrected partial correlation with exam status and psychological as well as sleep-related variables (PSS10, state anxiety, trait anxiety, awakening time, sleep duration and sleep quality) controlled showed that AUCg had a significantly positive association with alpha asymmetry score at frontocentral sites (see Fig. 1, partial *r* = 0.306 (95% CI [−0.004–0.573]), SE: 0.148, *p* = 0.034).

As an explorative analysis, the relationship between CAR and δ power (at Fz, FCz and Cz electrodes) was calculated. There was a trend of negative relationship between AUCg and δ power at Cz (*r* = −0.229, *p* = 0.093). This trend did not exist anymore with exam status and psychological as well as sleep-related variables (PSS10, state anxiety, trait anxiety, awakening time, sleep duration and sleep quality) controlled (partial *r* = −0.215 (95% CI [−0.561–0.128]), SE: 0.179, *p* = 0.142). There were no significant correlations between AUCi and δ power (|*r*|_*s*_ = 0.003–0.147, *p_s_* > 0.10).

## Discussion

We investigated the relationship between the CAR and lateralized frontocentral cortical activity under resting state. In general, our participants had a typical cortisol response after awakening, as indicated by the cortisol levels increased from awakening to peak level at 15–30 min after awakening, and then declined at 60 min after awakening. The results showed that cortisol within the first hour after awakening in the morning (AUCg) was positively associated with alpha asymmetry score at frontocentral cortical region but not attention-related δ power under rest even after controlling psychological (exam status, PSS10, state anxiety and trait anxiety) and diurnal pattern (awakening time, sleep duration and sleep quality) confounders.

As predicted, participants with higher total cortisol secretion in 1 h after awakening as reflected by AUCg also had relatively left-lateralized frontocentral cortical activity under resting state. The predictive and reactive control theory proposed that left hemisphere is more involved in self-determined, future-directed behavior and proactive coping, and the right hemisphere is more associated with externally-guided and reactive or avoidant coping (Tops et al., 2017). Thus, our result suggested that the higher CAR is associated with more active coping indicated by more left-lateralized frontocentral activity. In line with this, the higher CAR was linked to problem-focused rather than emotional coping style (Gilbert et al., 2017), less amount of aggressive behavior in a provocation task (Bohnke et al., 2010), lower levels of induced self-related negative emotional processing and more improvement of mood after induced distraction (Kuehner, Holzhauer & Huffziger, 2007). Furthermore, Quevedo et al. (2017) revealed that the higher CAR was related with larger activity in dACC during negative self-descriptors, and they explained that a heightened arousal system (higher dACC activity) may increase preparedness for negative events as indicated by elevated levels of CAR. The result in the current study extended the association between the CAR and coping measured from behavioral tasks to resting state brain function.

In our study, the higher CAR as a trait was associated with a larger alpha asymmetry score thus more left-lateralized alpha asymmetry in the frontocentral regions in resting state. Interestingly, the resting state functional magnetic resonance imaging study revealed the similar results in healthy male students, in which the larger CAR in the morning was correlated with stronger intrinsic functional connectivity strength of medial prefrontal cortex (mPFC) as well as stronger positive mPFC connectivity with regions in the default mode network in the afternoon. Based on these, the authors proposed that “higher CAR might be a predictive biomarker of brain activity” (Wu et al., 2015). Our result, however, contradicted with Hewig et al. (2008) that the higher CAR was associated with greater right frontal cortical activity under resting state when participants face an acute naturalistic stressor. These inconsistent findings might result from the different characteristics between Hewig et al. (2008) and our experiment, like time of stressor (acute vs. distant), gender (mainly female vs. male) and EEG topography (lateral frontal vs. frontocentral). Furthermore, as the authors mentioned, they measured only 1 day of the CAR which might be influenced to a large extent by situational factors, neither did they control any psychosocial variables and diurnal activity change which have demonstrated to be crucial for the CAR (Clow et al., 2010; Fries, Dettenborn & Kirschbaum, 2009). To increase the stability of CAR, the current study focused on trait measures of the CAR by collecting the morning samples on two consecutive days (Hellhammer et al., 2007). Participants were instructed to avoid staying up late and wake up between six and eight o’clock in the morning to minimize the diurnal activity change on CAR. Furthermore, we further controlled the psychological (exam status, PSS10, state anxiety and trait anxiety) and diurnal pattern (awakening time, sleep duration and sleep quality) in the partial correlation analysis, and the relationship between the CAR and alpha asymmetry score still existed.

It’s worthwhile to mention that no causality can be attributed to the result of the relationship between the higher CAR and more left-lateralized frontocentral cortical activity under resting state. However, there are three possible interpretations of this result. One possibility is that the CAR may exert a slow genomic effect on brain activity. Several animal and human studies have revealed that cortisol exerts time-dependent effects on neurobiological processes. After acute stress, the rapid increase of cortisol enhanced limbic salience processing, while impairing PFC executive function by fast non-genomic effect; several hours after stress and cortisol peak, slower long-lasting genomic corticosteroid actions develop to restore PFC functions (Henckens et al., 2010, 2011; Joels et al., 2008; Qin et al., 2009). In our study, the natural peak of circadian cortisol (i.e., CAR) instead of laboratory manipulated cortisol peak was associated with brain activity under resting state. The results suggested that the higher activity of HPA axis in the early morning is associated with more left lateralized brain activity, which might mediate by slow genomic corticosteroid effect. The second explanation is that elevated cortisol might be mood protective, helping individuals cope with the emotional load of situations by buffering negative emotional responses (Hoyt et al., 2016). For example, Het & Wolf (2007) found that cortisol-treated participants reported significantly less negative affect after stress exposure than that of placebo-treated subjects. Meanwhile, it has been noted that normal elevation of morning cortisol serves to mobilize energy resource storage, increase interest in exploration, and promote attention focusing and consolidation in the learning process (Gunnar & Vazquez, 2001). Therefore, the current findings support the boost hypothesis of CAR, that is, appropriately exploiting energy resources (the higher CAR) might facilitate more positive emotion, as well as more active approach tendency which is characterized as more left-lateralized frontocentral activity. The third explanation underlying the relationship between the higher CAR and left-lateralized frontocentral activity is that a common factor might shape both the CAR and resting state frontocentral activity. On the one hand, ambulatory cortisol levels were found to be related with appraisal. Specifically, the CAR increased less when stressors were appraised as more stressful and uncontrollable (Gartland et al., 2014), while it increased more when individuals use problem solving as an emotion regulation strategy (Gilbert et al., 2017; Jackson et al., 2003). On the other hand, left-lateralized frontal activity is involved in positive emotion processing, faster recovery from the stressful event (Jackson et al., 2003; Urry et al., 2004). Those who came to the laboratory might appraise the novel laboratory environment as controllable stressor and have a more active engagement with rather than conservation/withdrawal response to the experiment, and this active coping may create a link between the increased CAR and more left-sided cortical activity.

Only AUCg was associated with left-lateralized frontocentral activity, but no significant correlation was found between the AUCi and alpha asymmetry score. AUCg is the total area under the curve with respect to ground in 1 h after awakening which “takes into account both sensitivity (the difference between the single measurements from each other) and intensity (the distance of these measurements from group)” (Fekedulegn et al., 2007). AUCi is the area under the curve with respect to the baseline measurement and more focuses on the dynamic change in 1 h after awakening and is more associated with HPA axis’s sensitivity (Clow et al., 2010). Furthermore, AUCg (but not AUCi) has been demonstrated to be associated with the diurnal cortisol pattern for the following 12 h, which suggests that AUCg and AUCi might be regulated by different neuroendocrine pathways (Edwards et al., 2001). Several previous studies also found that the AUCg rather than AUCi is linked with mental and physical health (Lamers et al., 2013; Rane et al., 2012), working memory performance (Moriarty et al., 2014) and connectivity strength between mPFC and default mode network under resting state (Wu et al., 2015). These findings together with ours suggest that AUCg and AUCi reflect different brain mechanisms, and thus need to be explored separately.

Our research has some limitations that need to be addressed. First of all, our EEG data were based on recordings from a single session only. Although previous studies showed that there was high internal consistency reliability of the EEG asymmetry measures (Tomarken et al., 1992; Urry et al., 2004), we still emphasize that collecting data more than one single session would help improve the stability of the results. Secondly, we only recruited male university students, therefore, the results we found here might not be able to be generalized to females or non-university student samples.

## Conclusions

In conclusion, our study demonstrates that the total cortisol secretion after awakening as indexed by AUCg is associated with active coping as indexed by lateralized cortical activity in the frontocentral region. The relationship between natural cortisol peak (i.e., CAR) and alpha asymmetry might be mediated by long-term genomic corticosteroid actions. Future studies should include female individuals and more resting state EEG sessions to replicate this result.

## Supplemental Information

10.7717/peerj.7059/supp-1 Supplemental Information 1 Raw dataset. The relationship between CAR and alpha asymmetry.

10.7717/peerj.7059/supp-2 Supplemental Information 2 Questionnaires (Chinese version). The questionnaires we used in the study were already translated and validated in Chinese population.

## Additional Information and Declarations

### Data Availability

The following information was supplied regarding data availability: The raw measurements are available as a Supplemental File.
