# Supplementary material for: Associations between cortisol awakening response and resting electroencephalograph asymmetry
Source: PeerJ. 2019 Jun 3;7:e7059. doi: 10.7717/peerj.7059 (PMC6553442; doi:10.7717/peerj.7059)
Supplement: Supplemental Information 2 — The questionnaires we used in the study were already translated and validated in Chinese population. [file peerj-07-7059-s002.zip › questionnaires/PSS10.docx]

**知觉心理压力量表(CPSS)**

下面我们将询问近一个月来您对某些事件的感受或想法。其中的有些问题表面上看起来似乎相似，但是它们之间实际上却存在着差别，应区别对待。回答时最好不要去试图计算具体的次数,只需尽快给出一个合乎实际的估计。请在下面每个问题的五个选择项中选一项作为您的回答，并在相应的方框内打“√”。

| 条目 | 从来没有 | 极少有 | 有时有 | 经常有 | 总是有 |
| --- | --- | --- | --- | --- | --- |
| 1、过去一个月中，你为一些预料之外事情的发生而感到不安？ |  |  |  |  |  |
| 2、过去一个月中，你感觉到不能控制生活中的重要事情？ |  |  |  |  |  |
| 3、过去一个月中，你感觉到紧张和压力？ |  |  |  |  |  |
| 4、过去一个月中，你感觉到有信心能够处理好自己的问题？ |  |  |  |  |  |
| 5、过去一个月中，你感觉到事情在按照自己的意愿在发展？ |  |  |  |  |  |
| 6、过去一个月中，你发现不能应付自己所必须要做的事情？ |  |  |  |  |  |
| 7、过去一个月中，你能够解决生活中令人不快的事？ |  |  |  |  |  |
| 8、过去一个月中，你感觉到能够掌控自己生活中的事情？ |  |  |  |  |  |
| 9、过去一个月中，你为发生了一些自己无法控制的事情而感到气愤？ |  |  |  |  |  |
| 10、过去一个月中，你感觉到问题在不断地累积而不能得到解决？ |  |  |  |  |  |

Perceived Stress Scale

The questions in this scale ask you about your feelings and thoughts during the last month. In

each case, you will be asked to indicate by circling how often you felt or thought a certain way.

0 = Never 1 = Almost Never 2 = Sometimes 3 = Fairly Often 4 = Very Often

1. In the last month, how often have you been upset

because of something that happened unexpectedly?.................................. 0 1 2 3 4

2. In the last month, how often have you felt that you were unable

to control the important things in your life? .................................................. 0 1 2 3 4

3. In the last month, how often have you felt nervous and “stressed”? ............ 0 1 2 3 4

4. In the last month, how often have you felt confident about your ability

to handle your personal problems? ............................................................. 0 1 2 3 4

5. In the last month, how often have you felt that things

were going your way?.................................................................................. 0 1 2 3 4

6. In the last month, how often have you found that you could not cope

with all the things that you had to do? ......................................................... 0 1 2 3 4

7. In the last month, how often have you been able

to control irritations in your life?................................................................... 0 1 2 3 4

8. In the last month, how often have you felt that you were on top of things?.. 0 1 2 3 4

9. In the last month, how often have you been angered

because of things that were outside of your control?................................... 0 1 2 3 4

10. In the last month, how often have you felt difficulties

were piling up so high that you could not overcome them? ......................... 0 1 2 3 4
