# Supplementary material for: Associations between cortisol awakening response and resting electroencephalograph asymmetry
Source: PeerJ. 2019 Jun 3;7:e7059. doi: 10.7717/peerj.7059 (PMC6553442; doi:10.7717/peerj.7059)
Supplement: Supplemental Information 2 — The questionnaires we used in the study were already translated and validated in Chinese population. [file peerj-07-7059-s002.zip › questionnaires/sleep.docx]

**Instruction about saliva collection**

The related chemical component will be analyzed though saliva collection in order to explore the individual psychophysiological response. To protect the participants’ privacy, the data will be saved safely and properly. The instructions are as follows:

1. Keeping at least 7 hours of sleep the night before saliva collection， and waking up between 6 to 8 am (wake up naturally or awakened by clock).
2. **Please collect the first saliva sample immediately after you wake, then write down the time right away on log 2. This is very important, otherwise this sample wouldn’t be useful. We will also record your waking time and sampling time with technological method.**
3. Sampling method: Fetch a tube and open the top lid. Then take out the cotton and put it in your month (please do not touch it with your hand). Chewing for 2 minutes (please do not spit it out in the meantime), then carefully splitting it into the tube, and covering with the lid. **Writing down the specific time (T) and date (Date) on the label immediately**, and keeping in mind that do not mix the time between different tubes.

Note: The tube is produced by American Salimetrics company, and the tube and cotton are sterile and single use.

1. After the first saliva sample is collected, you can lie in bed or move freely in your dormitory/home and fill out three logs in the mean time. Other daily activities can be as usual, like reading books or magazine, listening to the radio, doing the makeup, face washing, going to the bathroom, conversing etc. Remember that the other two saliva samples will be collected 30 min and 45 min respectively after the first sample (the use of alarm clock to remind is recommended), and write down your specific sampling time. Then take a picture of the label every time you finish sampling.
2. During the 45 minutes (i.e., the sampling period): although your daily activity can be as usual, please do not nap or go back to sleep; you can stay in bed or get out of bed, but you must stay sober though you may still be a little sleepy. To avoid contamination of saliva, please do not brush or floss your teeth, smoke, drink any beverage (except water), eat, or take medicine before completion of saliva sampling in 45 minutes; do not exercise in the meantime;
3. After you finish the sampling procedure, put the saliva tubes in the refrigerator or in the shade, and bring them back to the laboratory when you come to do the experiment in the afternoon.
4. If you have any question, please contact: 06#######

I have read all the above notice and will comply with it (making duplicate of this questionnaire, one for the participant and one for the experimenter).

Signature:

Please collect the first sample immediately after you wake

Time you wake up:

How many hours you slept last night:

1. How did you sleep last night

1 2 3 4 5

Very poorly very well

1. Did you feel refreshed upon waking

1 2 3 4 5

Not at all completely

1. How deeply did you sleep last night

1 2 3 4 5

Lightly very deeply

1. Did you sleep for the entire time allocated for sleep

1 2 3 4 5

Woke up much earlier slept for the whole night

1. How easy was it for you to wake up

1 2 3 4 5

Very easy very difficult

1. How easily did you fall asleep last night

1 2 3 4 5

Very easily very difficult

1. How many dreams did you have last night

1 2 3 4 5

No dreams many dreams
