# Supplementary material for: Associations between cortisol awakening response and resting electroencephalograph asymmetry
Source: PeerJ. 2019 Jun 3;7:e7059. doi: 10.7717/peerj.7059 (PMC6553442; doi:10.7717/peerj.7059)
Supplement: Supplemental Information 2 — The questionnaires we used in the study were already translated and validated in Chinese population. [file peerj-07-7059-s002.zip › questionnaires/state_trait_anxiety_inventory.doc]

**状态－特质焦虑问卷（STAI）**

指导语：下面列出的是一些人们常常用来描述他们自己的陈述，请阅读每一个陈述，然后在右边适当的圈上打勾来表示你**现在**最恰当的感觉，也就是你此时此刻最恰当的感觉。没有对或错的回答，不要对任何一个陈述花太多的时间去考虑，但所给的回答应该是你现在最恰当的感觉。

|  | 完全  没有 | 有些 | 中等  程度 | 非常  明显 |
| --- | --- | --- | --- | --- |
| 1. 我感到心情平静 | ① | ② | ③ | ④ |
| 2. 我感到安全 | ① | ② | ③ | ④ |
| 3. 我是紧张的 | ① | ② | ③ | ④ |
| 4. 我感到紧张束缚 | ① | ② | ③ | ④ |
| 5. 我感到安逸 | ① | ② | ③ | ④ |
| 6. 我感到烦乱 | ① | ② | ③ | ④ |
| 7. 我现在正烦恼，感到这种烦恼超过了可能的不幸 | ① | ② | ③ | ④ |
| 8. 我感到满意 | ① | ② | ③ | ④ |
| 9. 我感到害怕 | ① | ② | ③ | ④ |
| 10. 我感到舒适 | ① | ② | ③ | ④ |
| 11. 我有自信心 | ① | ② | ③ | ④ |
| 12. 我觉得神经过敏 | ① | ② | ③ | ④ |
| 13. 我极度紧张不安 | ① | ② | ③ | ④ |
| 14. 我优柔寡断 | ① | ② | ③ | ④ |
| 15. 我是轻松的 | ① | ② | ③ | ④ |
| 16. 我感到心满意足 | ① | ② | ③ | ④ |
| 17. 我是烦恼的 | ① | ② | ③ | ④ |
| 18. 我感到慌乱 | ① | ② | ③ | ④ |
| 19. 我感觉镇定 | ① | ② | ③ | ④ |
| 20. 我感到愉快 | ① | ② | ③ | ④ |

指导语：下面列出的是人们常常用来描述他们自己的一些陈述，请阅读每一个陈述，然后在右边恰当的圈内打勾，来表示你**经常**的感觉。没有对或错的回答。不要对任何一个陈述花太多的时间去考虑，但所给的回答应该是你平常所感觉到的。

|  | 几乎没有 | 有些 | 经常 | 几乎总是如此 |
| --- | --- | --- | --- | --- |
| 21. 我感到愉快 | ① | ② | ③ | ④ |
| 22. 我感到神经过敏和不安 | ① | ② | ③ | ④ |
| 23. 我感到自我满足 | ① | ② | ③ | ④ |
| 24. 我希望能像别人那样高兴 | ① | ② | ③ | ④ |
| 25. 我感到我像衰竭一样 | ① | ② | ③ | ④ |
| 26. 我感到很宁静 | ① | ② | ③ | ④ |
| 27. 我是平静的、冷静的和泰然自若的 | ① | ② | ③ | ④ |
| 28. 我感到困难一一堆集起来，因此无法克服 | ① | ② | ③ | ④ |
| 29. 我过分忧虑一些事，实际这些事无关紧要 | ① | ② | ③ | ④ |
| 30. 我是高兴的 | ① | ② | ③ | ④ |
| 31. 我的思想处于混乱状态 | ① | ② | ③ | ④ |
| 32. 我缺乏自信心 | ① | ② | ③ | ④ |
| 33. 我感到安全 | ① | ② | ③ | ④ |
| 34. 我容易做出决断 | ① | ② | ③ | ④ |
| 35. 我感到不合适 | ① | ② | ③ | ④ |
| 36. 我是满足的 | ① | ② | ③ | ④ |
| 37. 一些不重要的思想总缠绕着我，并打扰我 | ① | ② | ③ | ④ |
| 38. 我产生的沮丧是如此强烈，以致我不能从思想中排除它们 | ① | ② | ③ | ④ |
| 39. 我是一个镇定的人 | ① | ② | ③ | ④ |
| 40. 当我考虑我目前的事情和利益时，我就陷入紧张状态 | ① | ② | ③ | ④ |
